# One-year mortality in displaced intracapsular hip fractures and associated risk: a report of Chinese-based fragility fracture registry

**DOI:** 10.1186/s13018-018-0936-5

**Published:** 2018-09-14

**Authors:** Simon Kwoon-Ho Chow, Jiang-hui Qin, Ronald Man-Yeung Wong, Wai-Fan Yuen, Wai-Kit Ngai, Ning Tang, Chor-Yin Lam, Tak-Wing Lau, Kin-Bong Lee, Kwai Ming Siu, Sze-Hung Wong, Tracy Y. Zhu, Wing-Hoi Cheung, Kwok-Sui Leung

**Affiliations:** 1 Department of Orthopaedics and Traumatology, Prince of Wales Hospital, The Chinese University of Hong Kong, 5/F, Clinical Sciences Building, Shatin, New Territories, Hong Kong, SAR China; 2 Department of Sports Medicine and Adult Reconstructive Surgery, Drum Tower Hospital, School of Medicine, Nanjing University, Nanjing, China; 3 Department of Orthopaedics and Traumatology, North District Hospital, Sheung Shui, Hong Kong; 4 Department of Orthopaedics and Traumatology, Tuen Mun Hospital, Tuen Mun, Hong Kong; 5 Department of Orthopaedics and Traumatology, Queen Mary Hospital, Pok Fu Lam, Hong Kong; 6 Department of Orthopaedics and Traumatology, Queen Elizabeth Hospital, Jordan, Hong Kong; 7 Department of Orthopaedics and Traumatology, Princess Margaret Hospital, Kwai Chung, Hong Kong; 8 Department of Acute and Rehabilitative Orthopaedics and Traumatology, Caritas Medical Centre, Sham Shui Po, Hong Kong

**Keywords:** Fragility Fracture Registry, Intracapsular fragility hip fracture, One-year mortality, Risk factors

## Abstract

**Background:**

The purpose of this registry-based retrospective study was to investigate the risk factors related to one-year mortality in displaced intracapsular fragility hip fracture patients.

**Methods:**

Patients were screened from the Fragility Fracture Registry. Inclusion criterion was displaced intracapsular hip fracture patients with atypical or pathological fractures excluded. One-year mortality was investigated against risk factors including age, gender, past medical history, pre-fracture mobility (PFM), pre-operation ASA grade, delayed surgery over 48 h, post-surgical complications, and length of stay at acute orthopedic ward (LOS).

**Results:**

A total of 1050 patients were included for further analysis. Gross one-year mortality was 14.9%. One-year mortality was significantly higher in patients who received non-operative treatment and those who received surgery but delayed over 48 h after admission (both *p* <  0.001). Male gender (OR = 2.708), advanced age (OR = 1.359), higher risk ASA grades (III to V) (OR = 1.990), past history of gastrointestinal disease (OR = 1.671), and renal impairment (OR = 1.984) were related to higher one-year mortality. The mortality of patients in PFM grade 3 and LOS group 3 was significantly higher (OR = 2.240 and 1.722, respectively).

**Conclusions:**

Higher age, male gender, past gastrointestinal disease and renal impairment, ASA grade over 3, indoor confined pre-fracture ambulatory, and stay at hospital over 15 days were risk factors related to higher one-year mortality in surgically treated displaced intracapsular hip fracture patients. A multi-disciplinary approach is advised to patients identified with these risks factors and co-managed by orthopedic surgeons, geriatricians, and fracture liaison nurses.

## Background

Fragility fracture is a kind of fracture that occurs as a result of a low-energy trauma, such as a fall from a standing height or less, or even no identifiable trauma. It is the main adverse consequence of osteoporosis [1]. Osteoporosis- related fragility hip fracture is a worldwide public health problem, as it is significantly associated with morbidity and mortality [2, 3]. It causes an increasing burden on the healthcare system especially in the aging society [4, 5]. Meanwhile, the incidences of osteoporosis and fragility fractures vary globally. According to Cooper’s study, around 30% of the fragility hip fracture occurring worldwide were arisen in Asian populations and the incidence rates still keep rising predominately among Chinese and Malay subsets of the population [6]. Interestingly, the age-adjusted incidences in Hong Kong and Singapore increased from 1960s to 1980s, synchronized with the modernization of the society [7, 8]. This suggests that urbanization with contemporary changes in physical activities and nutrition may contribute to the increasing incidences of fragility hip fractures [6]. As the urbanization process predominantly happens in Asia especially in Mainland China, a study of age-related, Chinese-based hip fracture in a developed Chinese society is of intriguing clinical implications.

Although the age-adjusted incidences of fragility hip fractures have become stable as healthcare quality improves [6], the gross incidences of fragility hip fractures keep steadily increasing because the society keeps aging and the mortality rate of these fractures was found to be generally high [9] . Compared to the annual 4% increase of mortality in hip fracture patients [10], the high mortality rate in the first year after fracture made this period most critical. Several factors have been identified related to the mortality of patients after surgery, such as increased age, male gender, ASA (American Society of Anesthesiologists) grade, types of surgery patients received, and walking ability before fracture [11, 12]. However, the results were controversial. For example, male gender was considered not related to the one-year mortality in the patients over 75 years old [11] but some other studies found it a major risk factor [13, 14]. In order to understand some population-specific risk factors that may contribute to increased mortality, a Fragility Fracture Registry was therefore established [9]. The objective of this study was to investigate the risk factors related to one-year mortality in displaced intracapsular fragility hip fracture patients, in which age, gender, pre-fracture mobility status, ASA grade, and length of stay at acute orthopedic ward were evaluated.

## Methods

Patients were retrospectively screened from the Fragility Fracture Registry (FFR, www.ffr.hk) database, which collected data of 2914 cases of fragility hip fracture patients who were admitted during 2012 to six major public hospitals under the management of the Hospital Authority of Hong Kong (www.ha.org.hk) [9]. Ethical approvals were obtained from the Research Ethics Committee (REC) of all six hospitals. Consent from subjects was not required. The study was done in accordance with the principles outlined in the Declaration of Helsinki. All patients fit the definition of fragility fracture as mentioned above [1] with age not less than 50 years old who sustained a hip fracture falling from standing height as previously described [9]. Only patients with displaced intracapsular hip fracture were included in this study. Cases of atypical or pathological fractures were excluded. Information including age, gender, pre-fracture medical history, previous fracture history, pre-fracture mobility status (PFM), pre-operation ASA (American Society of Anesthesiologists) grade, type of operation, surgical status at 48 h after admission, length of stay at acute orthopedic ward (LOS), and post-surgical complications were recorded.

The pre-fracture medical history record including previous episodes of arthritis, osteoporosis, cerebrovascular disease, dementia, depression, diabetes mellitus, gastrointestinal diseases, heart diseases, hypertension, hypotension, renal impairment, respiratory diseases, Parkinson’s disease, visual impairment, and other miscellaneous options. The post-surgical complications recorded the occurrence of deep vein thrombosis, wound infection, postoperative delirium, pressure sore, retention of urine, and other miscellaneous options. Time-to-surgery was calculated from the time of first presentation in hospital to the time of operation.

According to the ambulation status before fracture, PFM is divided into 3 grades: grade 1 (fully ambulatory), grade 2 (ambulatory with aids), and grade 3 (indoor confined).

According to the ASA score, patients were divided into 2 groups. The lower risk group (ASA grade I and II) included patients with healthy or mild systemic disease. The higher risk group (ASA grade III to V) included patients with severe non-incapacitating systemic disease and severe incapacitating systemic disease with a constant threat to life. No patient with ASA grade V was present in this study.

The impact of LOS on mortality of fragility hip fracture was studied extensively, but the grading standards vary among different studies [15, 16]. In this study, we consider LOS from the 25 percentiles (6.68 days) to 75 percentiles (15.09 days) as normal, the first quadrant as early-discharge patient and the last quadrant as delay-discharge patient. According to this standard, the patients were divided into 3 groups: LOS 1 (≤ 7 days); LOS 2 (7–15 days); LOS 3 (> 15 days).

### Statistical analyses

All data were managed using Research Electronic Data Capture (REDCap; Vanderbilt University) hosted in The Chinese University of Hong Kong [17]. Independent two-sample *t* test and Pearson’s Chi-square test were performed to compare age, gender, PFM, LOS, and one-year mortality between patients who received non-operative and surgical treatments. Potential risk factors related to the post-fracture one-year mortality were first screened by using Pearson’s Chi-square test (univariate). Risk factors including gender, age, PFM, LOS, ASA grade, delayed surgery, past medical history of respiratory disease, heart disease, hypertension, gastrointestinal disease, renal impairment, and post-surgical wound infection were further investigated by using logistic regression (multivariate). As delayed surgery was significantly associated with LOS and ASA grade, it was further excluded from the model to avoid collinearity. Female gender, PFM grade 1, low risk ASA group, and LOS group 2 were set as reference groups, respectively, and odds of mortality associated with age was presented as per decade increase. All statistical analyses were performed by using SPSS statistical software version 20.0 (IBM, NY, USA). A *p* value less than 0.05 was considered as statistically significant.

## Results

One thousand and sixty-one patients were captured during the study period. The majority of fractures (965 cases, 91%) were caused by fall. The leading causes included slip and fall (73.4%), fall from furniture (8.2%), and tripped by obstacles (5.8%). Eleven patients were excluded from the study due to missing data (one without one-year mortality, one without type of treatment, six without PFM, and three without LOS).

A total of 1050 patients (574 left hip fracture and 476 right hip fracture) were included for analysis in this study, among which 94.3% received surgical treatment and 5.7% received non-operative treatment. Table 1 shows age, gender, PFM, LOS, and one-year mortality of the patients by non-operative and surgical treatments. The gross one-year mortality of displaced intracapsular hip fracture was 14.9%, and mortality was significantly higher in the patients who received non-operative treatment than those who received surgical treatment (53.3% vs. 12.5%, *p* <  0.001). Age did not differ significantly between non-operative and surgical groups (*p* = 0.25), while the patients who received non-operative treatment were significantly more likely to be male (*p* = 0.028), in PFM grade 3 (*p* = 0.001), and have a longer LOS (*p* = 0.012). Previous episodes of cerebrovascular accidents (48.3% vs. 21.5%, *p* < 0.001) and heart diseases (58.3% vs. 29.2%, p < 0.001) were found significantly more common in the patients who received non-operative treatment.

**Table 1:** Demographic data on patients in the conservative and surgically treated group

Demographic variables		Conservative	Surgical
Number of patients		60 (5.7%)	990 (94.3%)
Gender	Male	26 (43.3%)	288 (29.1%)
	Female	34 (56.7%)	702 (70.9%)
Age, years	Mean ± SD	82.5 ± 10.0	81.1 ± 8.9
	Range	58–100	50–104
Pre-fracture mobility (PFM)	Grade 1	16 (26.7%)	399 (40.3%)
	Grade 2	22 (36.7%)	431 (43.5%)
	Grade 3	22 (36.7%)	160 (16.2%)
Length of stay (LOS)	Quadrant 1	24 (40.0%)	277 (28.0%)
	Quadrant 2	22 (36.7%)	467 (47.2%)
	Quadrant 3	6 (10.0%)	185 (18.7%)
	Quadrant 4	8 (13.3%)	61 (6.2%)
One-year mortality		32 (53.3%)	124 (12.5%)

Analyses were performed to identify risk factors for one-year mortality in the patients who received surgical treatment. Ten patients were excluded from the analyses due to lack of ASA grade. Analyses were performed in 980 patients. A total of 870 patients (88.8%) received unipolar hemiarthroplasty, which was the most commonly used surgical procedure. Of these patients, 768 received uncemented and 102 received cemented hemiarthroplasty. The use of cement during hemiarthroplasty showed no effect on the one-year mortality (12.7% in cemented who died vs. 13.0% in uncemented patients, *p* = 0.94). Five hundred and eighty-two patients (59.4%) received surgery within 48 h after admission while surgeries were delayed in 398 patients (40.6%). The most common reason of delayed surgery was prolonged medical review or stabilization of the patients (250 patients, 62.8%). One-year mortality was significantly higher in the patients with delayed surgery (54.5% vs. 38.6%, *p* = 0.001).

Logistic regression was performed to identify the risk factors (gender, increased age of every 10 years, PFM, LOS, ASA grade, past medical history of respiratory disease, heart disease, hypertension, gastrointestinal disease, renal impairment, and post-surgical wound infection) of one-year mortality (Table 2). Time-to-surgery was excluded from logistic regression analysis to avoid collinearity because it was significantly correlated with LOS and ASA score (both *p* < 0.001). Male gender was a strong risk factor of one-year mortality, with odds of mortality almost tripled in males than in females (OR = 2.708, 95% CI 1.766–4.151, p < 0.001). One-year mortality significantly increased with age, with each decade increase in age associated with 35.9% increase in mortality (OR = 1.359, 95% CI 1.041–1.773, *p* = 0.024). Patients with higher risk ASA score group had significantly higher one-year mortality than those with lower risk ASA score (OR = 1.990, 95% CI 1.182–3.349, *p* = 0.010). Mortality did not differ significantly between patients with PFM grade 2 and grade 1 (*p* = 0.253), but was significantly higher in the patients with grade 3 than those with grade 1 (OR = 2.240, 95% CI 1.229–4.085, *p* = 0.008). One-year mortality was similar between the patients who were fully ambulatory and those were ambulatory with aids, while patients who were indoor confined had 2.2 times higher risk of one-year mortality. Compared to the patients of LOS group 2, one-year mortality of group 3 was significantly higher (OR = 1.772, 95% CI 1.075–2.758, p = 0.024), but no difference was observed between group 2 and group 1, suggesting that risk of one-year mortality was higher only when LOS was beyond 15 days. Previous history of gastrointestinal disease (OR = 1.671, 95% CI 1.084–2.575, *p* = 0.020) and renal impairment (OR = 1.984, 95% CI 1.201–3.277, *p* = 0.007) were significantly associated with increased one-year mortality. Past history of respiratory disease, heart disease, hypertension, and post-surgical wound infection did not show significant impact on one-year mortality (*p* = 0.069, 0.149, 0.517, and 0.129, respectively).

**Table 2:** Mortality risk for different risk factors including pre and post-operational assessments and medical history

Risk factors	Dead	Alive	OR	95% CI	*p* value
Number of patients	121	859	–	–	–
Gender					
Male	59 (48.8%)	227 (26.4%)	2.708	1.766–4.151	< 0.001
Female*	62 (51.2%)	632 (73.6%)	–	–	–
Age, years			1.359^#^	1.041–1.773	0.024
Mean ± SD	8.36 ± 0.80	8.08 ± 0.89			
Range	5.9–10.0	5.0–10.4			
Pre-fracture mobility					
Grade 2	58 (47.9%)	367 (42.7%)	1.352	0.806–2.270	0.253
Grade 3	34 (28.1%)	125 (14.6%)	2.240	1.229–4.085	0.008
Grade 1*	29 (24.0%)	367 (42.7%)	–	–	–
ASA Grade					
High risk (III to V)	97 (80.2%)	433 (50.4%)	1.990	1.182–3.349	0.010
Low risk (I and II)*	24 (19.8%)	426 (49.6%)	–	–	–
Length of stay (LOS)					
Group 1 (≤ 7 days)	21 (17.4%)	255 (29.7%)	0.683	0.385–1.211	0.192
Group 3 (> 15 days)	53 (43.8%)	191 (22.2%)	1.722	1.075–2.758	0.024
Group 2 (7–15 days)*	47 (38.8%)	413 (48.1%)	–	–	–
Past respiratory disease					
Yes	31 (25.6%)	217 (25.3%)	1.584	0.965–2.600	0.069
No*	90 (74.4%)	642 (74.7%)	–	–	–
Past heart disease					
Yes	31 (25.6%)	217 (25.3%)	1.382	0.891–2.145	0.149
No*	90 (74.4%)	642 (74.7%)	–	–	–
Past history of hypertension					
Yes	31 (25.6%)	217 (25.3%)	1.172	0.725–1.896	0.517
No*	90 (74.4%)	642 (74.7%)	–	–	–
Past gastrointestinal disease					
Yes	31 (25.6%)	217 (25.3%)	1.671	1.084–2.575	0.020
No*	90 (74.4%)	642 (74.7%)	–	–	–
Past renal impairment					
Yes	31 (25.6%)	217 (25.3%)	1.984	1.201–3.277	0.007
No*	90 (74.4%)	642 (74.7%)	–	–	–
Post-surgical wound infection					
Yes	66 (54.5%)	332 (38.6%)	1.920	0.826–4.460	0.129
No*	55 (45.5%)	527 (61.4%)	–	–	–

^#^Per decade increase in age

*Reference group

## Discussion

In this study, we investigated one-year mortality and its related risk factors in the patients with displaced intracapsular hip fracture in a Chinese-based population. We found that patients who received non-operative management had higher mortality than those who received surgical treatment. However, direct evaluation of pre-surgical health condition of these patients was not feasible due to lack of ASA scores in the non-operative treated patients, yet their poorer pre-fracture mobility and longer length of stay at acute orthopedic ward still indicated poorer general health condition and more in-hospital comorbidities. These patients also had higher incidence of previous episode of cerebrovascular accident and heart disease. A study on Korean population also reported associations between mortality and chronic illnesses including hypertension, diabetes, dyslipidemia, ischemic heart disease, and stroke [18]. Hence, poor general health condition was the major factor correlated to the patients’ selection of non-operative management, while age and gender showed no effect.

In the patients who received surgical treatment, higher mortality in male was observed. This result was in agreement with a similar previous study [14] that is also associated with advanced age similar to a previous study [19]. Previous studies found that male patients with hip fracture usually had poorer health status before surgery with worse ASA grades [13] and pre-existing cerebrovascular and pulmonary diseases were associated to more post-surgical comorbidities such as developing pneumonia and cardiac complications [13, 20]. However, in our multivariate logistic regression analyses, male gender remained significant after adjustment of ASA grade and past medical history of comorbidities, suggesting that, at least in part, male gender per se led to higher one-year mortality of hip fracture. However, due to different inclusion criteria and varied sample sizes among studies, direct comparison with previous studies must be taken with caution. It has also been reported that gender was not related to the mortality in hip fracture patients over 75 years old [11].

Pre-fracture mobility reflects the general condition of patient’s health and related to the outcomes of surgery. Previous studies reported that walking ability before hip fracture could predict greater long-term survival [12] and affect the short-term and long-term mortality in hip fracture patients [20]. Similar findings were observed in this study. Patients with poorer ambulatory status before fracture was found related to higher one-year mortality rate. Length of hospital stay was correlated to both short-term and long-term mortality in surgically treated hip fracture patients. Cohort studies from northern Europe showed that LOS of less than 10 days was associated to both better short-term and long-term survival [16, 21]. However, LOS of less than 5 days was found to be associated with reduced early mortality rates when comparing to LOS of 11–14 days in the USA [15]. Our study found that there was no difference in one-year mortality between early discharged patients with LOS less than 7 days and those with LOS between 7 to 15 days. On the other hand, LOS over 15 days was found correlated to increased short-term mortality in the USA [15] and higher one-year mortality in our study. In Denmark, prolonged LOS over 20 days was also associated with long-term mortality [21]. The lack of difference between short and intermediate LOS in this study may be due to a lack of interdisciplinary management. Since our current study also revealed that history of geriatric medical conditions like gastrointestinal and renal diseases substantially increased the risks of one-year mortality, and also shown by our previous report that only 3.5% of hip-fracture patients received geri-orthopedic co-management [9]. Furthermore, decreased muscle strength and vitamin D deficiency in geriatric hip fracture patients were also reported to increase mortality rates in the Spanish FONDA cohort [22]. Therefore, a multi-disciplinary management approach [23, 24] (by orthopedic surgeons, geriatricians, and fracture liaison nurses) is strongly advised to patients identified with these risks factors (in particular, pre-fracture mobility and previous medical conditions) in order to proactively work towards improving the survival rate after an intracapsular hip fracture event. More detailed investigation is suggested to dissect the relative risks in various medically compromised groups.

There are limitations in this study. The power of this study is limited to the intracapsular neck of femur fractures that generalization to hip fracture shall be taken with caution. DXA data were not available from the public healthcare system for further analysis. Cognitive status was closely related to the prognosis of patients with hip fracture [25, 26]. However, it was not analyzed in this study due to the relatively small number of patients who received cognitive function assessments, and the influence of medication to the fracture was not studied due to similar reasons.

## Conclusion

In the patients with displaced intracapsular hip fracture, one-year mortality was significantly higher in the patients who received non-operative treatment than those who received surgical treatment. Risk factors related to one-year mortality in surgically treated displaced intracapsular hip fracture patients included older age, male gender, ASA grade over 3, an indoor-confined pre-fracture mobility status, and stay at hospital over 15 days. A multi-disciplinary approach is advised to patients identified with these risks factors and co-managed by orthopedic surgeons, geriatricians, and fracture liaison nurses.

## Abbreviations

ASA: American Society of Anesthesiologists
CI: Confidence interval
FFR: Fragility Fracture Registry
LOS: Length of stay
OR: Odds ratio
PFM: Pre-fracture mobility
REDCap: Research Electronic Data Capture
